# THE VARIATION IN DAILY HABITUAL PHYSICAL ACTIVITY LINKS TO GLOBAL COGNITIVE FUNCTION IN OLDER ADULTS

**DOI:** 10.1093/geroni/igad104.2309

**Published:** 2023-12-21

**Authors:** Levi Ask, Brad Manor, On-Yee Lo

**Affiliations:** Hebrew SeniorLife, Boston, Massachusetts, United States; Hebrew SeniorLife/Harvard Medical School, Amherst, New Hampshire, United States; Hebrew SeniorLife/ Harvard Medical School, Boston, Massachusetts, United States

## Abstract

Being physically active is critical to the maintenance of physical and cognitive function into old age. Wearable sensors with the ability to monitor step counts have become popular, making it easier for individuals to track their physical activity levels from day to day. While average daily step count has traditionally been used to quantify physical activity, emerging evidence suggests that the magnitude of day-to-day variation in physical activity or other clinical outcomes may provide additional insight into health. This study aimed to investigate the relationship between the characteristics of habitual physical activity and global cognitive function in older adults. A secondary analysis from a pilot randomized controlled trial was conducted. Thirty-two older adults (79±7 y/o, 29 women) completed the Montreal Cognitive Assessment (MoCA) of global cognitive function at baseline (Total MoCA score: 25.0±2.9). Physical activity was tracked using a Fitbit wrist-worn activity tracker for two consecutive weeks with all participants having 10 complete days. Daily step count mean and standard deviation were calculated to quantify the daily average (4435.12±2768.88 steps) and daily variation (1671.05±1134.03 steps) in physical activity, respectively. After adjusting for age and sex, participants with greater daily variation in step counts exhibited better global cognitive function (r=0.52, p=0.02). In contrast, average daily steps did not significantly correlate with MoCA performance. Neither of these physical activity outcomes were significantly linked to any MoCA subscore. These results suggest that monitoring day-to-day step count variation may serve as a particularly sensitive indicator of global cognitive health in older adults.

